# COVID-19 Data Utilization in North Carolina: Qualitative Analysis of Stakeholder Experiences

**DOI:** 10.2196/29310

**Published:** 2021-09-02

**Authors:** Jenny Rees Patterson, Donna Shaw, Sharita R Thomas, Julie A Hayes, Christopher R Daley, Stefania Knight, Jay Aikat, Joanna O Mieczkowska, Stanley C Ahalt, Ashok K Krishnamurthy

**Affiliations:** 1 University of Iowa Iowa City, IA United States; 2 North Carolina State University Raleigh, NC United States; 3 Cecil G. Sheps Center for Health Services Research University of North Carolina at Chapel Hill Chapel Hill, NC United States; 4 Renaissance Computing Institute University of North Carolina at Chapel Hill Chapel Hill, NC United States

**Keywords:** qualitative research, interview, COVID-19, SARS-CoV-2, pandemic, data collection, data reporting, data, public health, coronavirus disease 2019

## Abstract

**Background:**

As the world faced the pandemic caused by the novel coronavirus disease 2019 (COVID-19), medical professionals, technologists, community leaders, and policy makers sought to understand how best to leverage data for public health surveillance and community education. With this complex public health problem, North Carolinians relied on data from state, federal, and global health organizations to increase their understanding of the pandemic and guide decision-making.

**Objective:**

We aimed to describe the role that stakeholders involved in COVID-19–related data played in managing the pandemic in North Carolina. The study investigated the processes used by organizations throughout the state in using, collecting, and reporting COVID-19 data.

**Methods:**

We used an exploratory qualitative study design to investigate North Carolina’s COVID-19 data collection efforts. To better understand these processes, key informant interviews were conducted with employees from organizations that collected COVID-19 data across the state. We developed an interview guide, and open-ended semistructured interviews were conducted during the period from June through November 2020. Interviews lasted between 30 and 45 minutes and were conducted by data scientists by videoconference. Data were subsequently analyzed using qualitative data analysis software.

**Results:**

Results indicated that electronic health records were primary sources of COVID-19 data. Often, data were also used to create dashboards to inform the public or other health professionals, to aid in decision-making, or for reporting purposes. Cross-sector collaboration was cited as a major success. Consistency among metrics and data definitions, data collection processes, and contact tracing were cited as challenges.

**Conclusions:**

Findings suggest that, during future outbreaks, organizations across regions could benefit from data centralization and data governance. Data should be publicly accessible and in a user-friendly format. Additionally, established cross-sector collaboration networks are demonstrably beneficial for public health professionals across the state as these established relationships facilitate a rapid response to evolving public health challenges.

## Introduction

In 2020, the World Health Organization declared the outbreak of COVID-19—a public health emergency of international concern [1]. First identified in Wuhan, China, the virus quickly became a global pandemic, with over 181 million recorded cases and 3.94 million deaths reported worldwide as of June 2021 [2]. As of June 2021, the United States had more than 33 million COVID-19 cases and more than 600,000 COVID-19 deaths [2]. At the time of this study (in June 2020), North Carolina public health workers witnessed the growing national crisis and felt a sense of urgency to respond due to a state average of 1859 new infections each week [3].

Almost two decades ago, the Centers for Disease Control and Prevention established preparedness and response guidance in response to the 2003 SARS outbreak [4]. This guidance was intended to inform future infectious disease emergencies and included 4 overarching themes: (1) the need for up-to-date local, national, and global data; (2) rapid and effective institution of control measures; (3) appropriate resources and decision-making structure; and (4) trained staff vital to swift and decisive implementation [5]. While these recommendations were intended to prepare the country to handle a pandemic, few were truly prepared for the exceptionally rapid and widespread impact of the COVID-19 virus. As COVID-19 continued to spread, policy makers and public health officials at every level were forced to recognize the severity of the virus and take action to mitigate the spread.

As news of this complex public health problem spread in early 2020, North Carolinians relied on data from local, state, federal, and global health organizations to increase their understanding of the pandemic and guide decision-making. We aimed to understand how organizations across the state were collecting, analyzing, and reporting COVID-19 data. We were interested in the sources of data, as well as its uses. Additionally, we asked how data were aggregated, centralized, and disseminated.

## Methods

### Study Design

We used an exploratory qualitative study design to investigate North Carolina’s COVID-19 data collection efforts [6-8]. In-depth interviews were used to gather information and document the evolution of North Carolina’s COVID-19 response, with a focus on gaining a better understanding of COVID-19 data sources; data collection and reporting protocols and objectives; data uses and dissemination; data aggregation and centralization; and COVID-19 testing.

### Recruitment

Key informants were identified as experts in their fields who were known to be involved with COVID-19–related data. Potential interviewees were identified through a series of steps that included project team discussions, external peer consultations, and internet-based searches. Prior to conducting interviews, the project team met to prioritize the list of potential interviewees based on their involvement in and proximity to COVID-19 data. A snowball sampling approach was utilized to recruit key informants beyond the initially identified expert group [9,10].

After identifying potential interview participants, we prioritized and randomly assigned interviews among the project team. The interviewers contacted their assigned interview participants via email to request an interview and explain the overall project aim—to understand how COVID-19 data are being collected and reported across the state. Interviewers identified themselves in the recruitment email as members of the research team led by the Renaissance Computing Institute at University of North Carolina Chapel Hill and funded by the North Carolina Policy Collaboratory. The recruitment email also included the interview questions.

The interviews were not intended to be statistically representative of the state, and the number of interviewees does not affect the integrity of data collected. However, we attempted to obtain coverage from all regions of North Carolina to account for geographic and demographic differences. Recruitment of interview participants ended once thematic saturation was reached in response data and no new topics emerged [11].

### Interviews

We developed a semistructured interview guide (Textbox 1), which included open-ended questions covering the topics of data sources, uses, and how data were aggregated and reported [12].

**Questions about data collection processes in North Carolina.**

When did you begin collecting COVID-related data?

What were your objectives when you started collecting data?

Has the objective evolved? In what ways?

What guidance, if any, have you received from other organizations?

What were the biggest barriers in your work?

What type of patient-level/individual data is your organization collecting?

What challenges have you experienced in collecting individual-level data?

How does your organization collect data on patient contact/contact tracing?

How are hospital capacities being reported?

How are hospital utilizations being reported?

How is comorbidity being addressed?

How are the results of data collection being reported up to NCDHHS?

How are COVID-19 diagnoses and outcomes being centralized?

What is the purpose of data models you use?

Is there data that you need, but don’t have, for your models to be more accurate?

How are decisions made by your organization regarding data accessibility and dissemination?

What are some ways in which data dissemination has informed on or positively impacted the state of the pandemic?

The interviews were conducted by 4 team members (JA, JOM, SCA, and AKK). Interviews were conducted in an informal conversational manner in which interviewees were assured of their expertise so that they felt comfortable in freely stating their views. The goal here was to gain the trust of the interviewee and foster an environment of power equality [12,13]. Interviewers practiced the techniques of active listening and used follow-up questions when needed for clarification to capture accurate and thorough data [14].

### Confidentiality

Interview participants were told of the voluntary nature of this project and verbal consent to record and transcribe responses for analyses was obtained prior to the start of the interview. Interview participants were informed that the recordings would be deleted after the conclusion of the study and would not be shared outside of the project team or used for any other projects in the future. Interviewers explained the aim of the research, and how interview responses would be used to inform a report describing the use of COVID-19 data in the state. Furthermore, interview participants were told that the content of the interview would be deidentified, and any information used in the report would not cite an interviewee by name unless permission was given voluntarily.

### Analysis

Interviews were recorded and transcribed via Zoom (Zoom Inc). Scribes attended each interview to transcribe in real time and subsequently reviewed and edited transcripts for accuracy using the recordings.

Transcribed data were imported and analyzed using NVivo qualitative data analysis software (versions 11 and 12; QSR International). Data were analyzed using a hybrid approach to content analysis, which is a suitable methodology for interview transcripts [15-17]. First, 2 qualitative analysts used the interview guide questions to deductively choose categories, which served as the basis of the codebook (eg, data uses, challenges) [18]. As such, some codes were defined beforehand from the interview guide, while the remaining codes were defined as they emerged during analysis. To increase validity, 3 team members who were knowledgeable and experienced in qualitative research methods independently reviewed the transcripts and developed inductive codes (eg, modeling, dashboards, data lags, data consistency) [15]. This approach allowed for themes to arise directly from the data. Themes were identified through the techniques of cutting and sorting, repetition, and similarities or differences [19]. Analysis team members set regular meetings to compare, review, and refine codes. Discrepancies in codes were resolved through discussion [20]. Emerging themes and coding memo notes were also shared and discussed as a group. As analysis progressed, the transcripts coded early in the process were reread to refine and recode in consideration of codes developed later as more interviews were completed and more data became available.

Rigor was ensured by (1) triangulating different sources of data (eg, key informant interviews, literature and grey literature review, and notes) [21]; (2) employing independent coding of transcripts and intercoder agreement; and (3) utilizing an iterative process in which data collection and analysis happened concurrently, allowing for data collection to end only once thematic saturation was observed (ie, no more interviews were required) [12].

## Results

### Interview Participants

The response rate for interview requests was 59% (41/69). Key informants (n=41) participated in a total of 29 in-depth videoconference interviews during the period from June through November 2020. Interview participants included hospital workers, academics, individuals from health research organizations, state health department employees, health educators, laboratory employees, and others (Table 1). In some instances, there were multiple interviewees from the same organization. When this occurred, we sought to identify interviewees with varying roles within the organization so that their relationships with and perspectives on the data were different and provided a comprehensive and robust data set. During these interviews, each interviewee was provided time to respond to each question, and their responses provided insight into their roles within the organization. Most interview participants had roles in collecting, analyzing, and reporting or modeling data. No compensation was offered for participation in interviews.

**Table 1:** Participants’ demographic information.

Characteristic			Value (n=41), n (%)
**Gender**			
	Male	22 (54)	
	Female	19 (46)	
**Relationship to COVID-19 data^a^**			
	Collects	34 (83)	
	Analyzes	40 (98)	
	Reports or models	34 (83)	
**Work environment**			
	Hospital	11 (27)	
	Academia	7 (17)	
	Health research organization	6 (15)	
	State health department	5 (12)	
	Health education center	4 (10)	
	Laboratory	3 (7)	
	Nonprofit research organization	3 (7)	
	Health care management	2 (5)	

^a^More than 1 category is possible; therefore, percentages do not add to 100%.

### COVID-19 Data Flow

**Figure 1:**
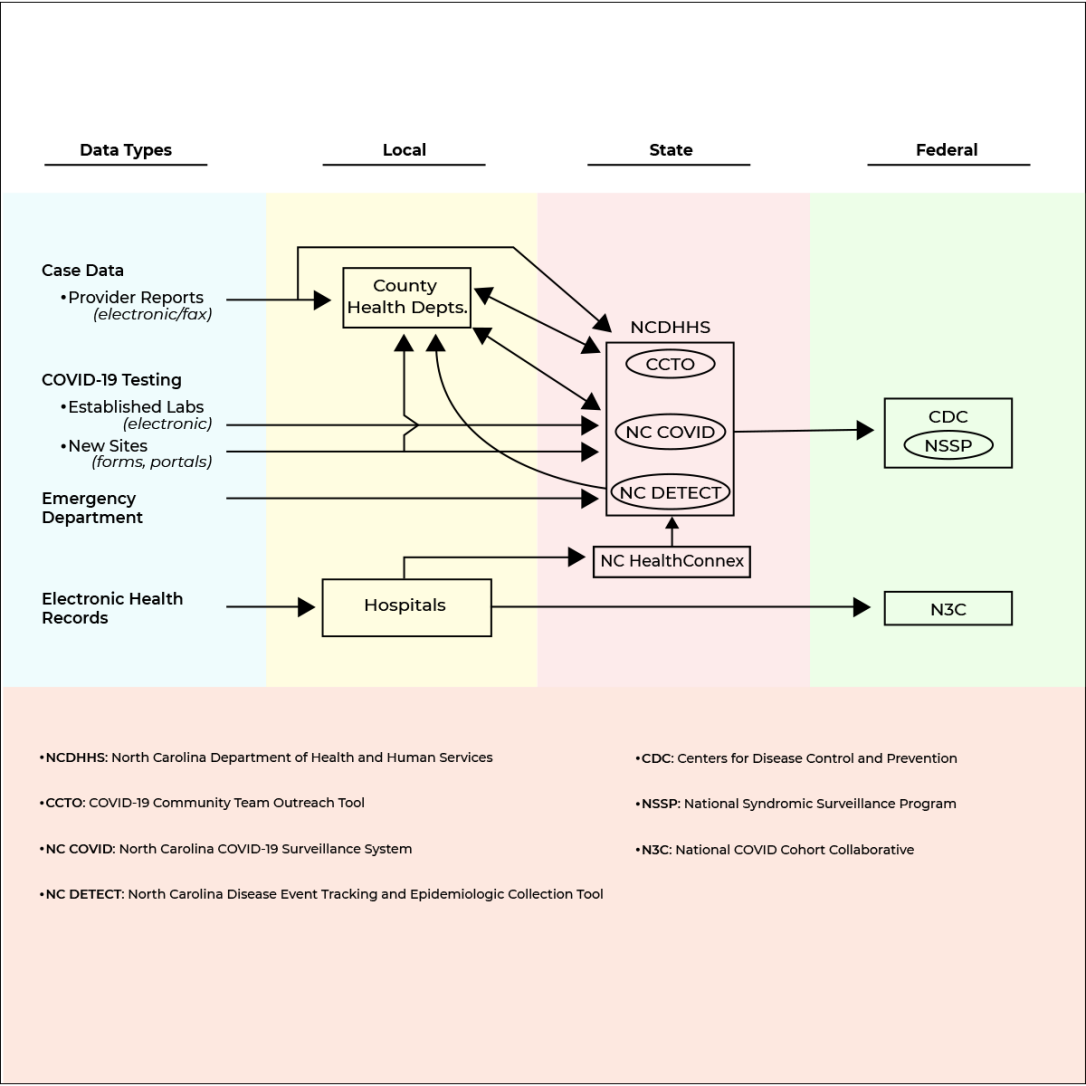
COVID-19 data flow in North Carolina in 2020.

Interviewees provided our research team with information regarding the flow of COVID-19 data across North Carolina (Figure 1). In North Carolina, COVID-19 data is generated from cases, COVID-19 testing, emergency departments, and electronic health records (EHRs).

Case data, or data from COVID-19 case investigations, are in the form of medical provider reports, sent both electronically and via fax to local health departments and the North Carolina Department of Health and Human Services (NCDHSS). The local health departments then have a 2-way flow of case data with NCDHHS’ COVID-19 Community Team Outreach Tool for tracing efforts, and NCDHHS’ COVID-19 Surveillance System.

COVID-19 testing data are gathered from established laboratories electronically and from new testing sites via forms and newly developed portals; the data are sent to local health departments and NCDHHS’ COVID-19 Surveillance System. COVID-19 tests are completed by private companies (eg, pharmacies, private laboratories) and public organizations (eg, county testing sites). As of December 2020, physicians, laboratories, and other health care providers in North Carolina were mandated to report COVID-19 test results, and key data fields (eg, patient, laboratory, and test data) have been identified [22].

Data from emergency departments are sent directly to NCDHHS’ North Carolina Disease Event Tracking and Epidemiologic Collection Tool [23], which, as the state syndromic surveillance system that has long been used by hospitals to report emergency department data electronically, then communicates these data to local health departments.

COVID-19 data from EHRs are sent from local hospitals to (1) the state’s health information exchange system (NC HealthConnex platform) and (2) the National COVID Cohort Collaborative. NC HealthConnex also sends this information on to NCDHHS.

Finally, all the COVID-19 data received by NCDHHS are then communicated at the federal level to the Centers for Disease Control and Prevention and the National Syndromic Surveillance Program.

### Data Collection Objectives

Most interview participants started collecting COVID-19–related data in mid to late March 2020. None of the interview participants indicated having a predetermined objective or established protocol to guide the data collection process, but all mentioned feeling compelled to take some action. A common initial objective for collecting COVID-19 data was the need to monitor hospital resource supply and utilization, including tracking intensive care unit volumes, negative pressure rooms, patients testing positive for COVID-19, and consumption rates for personal protective equipment. This evolved so that later more complex systems were in place to focus on hospitalizations and capacity.

Many interviewees noted their overall main objective in collecting COVID-19–related data remained unchanged since the start of the pandemic. Nonetheless, approaches were adapted as more was learned about the virus to reflect the broader community’s needs and overall response to the pandemic. Small adjustments in data collection were a direct result of state and federal mandates for COVID-19 data. A few ways in which data requests evolved included a departure from solely reporting the percentage of positive tests to now also requiring negatives as well as comparing asymptomatic and symptomatic positivity rates. According to interviewees this was an important development as, up until that time, data from hospitals and laboratories were only based on individuals testing positive, meaning when a patient tested negative, they would no longer be a part of hospital-based reporting. Furthermore, state mandates in the summer added order-based questions to reporting, which included indicating race and ethnicity and whether patients were symptomatic or pregnant. Others noted a shift in requirements for patient types and counts (ie, a shift from overall inpatient counts to COVID-19–related deaths). As a result of these changes, some interview participants mentioned the need to retrospectively look at data not initially reported in order to understand trends over time.

### Data Sources

The primary source of COVID-19 data used most by interview participants was their health care facilities’ EHR systems. One type of EHR system—EPIC—was mainly utilized. One interview participant collected qualitative primary data through surveys and interviews to gain the perspective of local government leaders on how COVID-19 was affecting their community. Another group used surveys to determine how to modify people’s behavior to mitigate spread of the virus.

Almost all interview participants reported using COVID-19 data available from secondary data sources. Publicly accessible secondary sources used by many of the interview participants included The New York Times COVID-19 data on GitHub [24], The News & Observer [25], and the WRAL website [26]. The New York Times was mentioned by multiple interview participants who expressed its importance in understanding regional differences and time trends in the county-level data.

One interview participant noted that insurance claims data from BlueCross, BlueShield, or Medicaid was not a good source because of data lag, which is the difference in time from when an event happens or is reported to when the relevant data becomes available for use. Insurance claim data, which can provide insight on individual-level interactions with health systems, often lag by 3 to 6 months [27]. Others mentioned using secondary data sources made available by NCDHHS, such as the North Carolina Disease Event Tracking and Epidemiologic Collection Tool and the COVID-19 Surveillance System.

Additional secondary sources utilized for COVID-19 data activities included SafeGraph [28], scientific literature, annual demographic poll data, PolicyMap [29], and mobility and weather data found on the internet. One interviewee mentioned scanning websites for manufacturer press releases to remain informed on ventilators and other personal protective equipment.

### Uses of COVID-19–Related Data

#### Dashboards

The most common use of COVID-19–related data, mentioned by approximately one-third of interview participants, was the creation of dashboards. Web-based dashboards can serve as a user-friendly tool to help policy makers, public health professionals, and the public visualize COVID-19 data in real time. Some interview participants developed dashboards in response to requests from NCDHHS to help predict cases and provide the public and other health professionals with up-to-date information. Others took it upon themselves to make data that was already available more useful to the public so that they could have a better understanding of their current risk. Interview participants reported using dashboards internally within organizations as well as externally and across organizations. Dashboards incorporated data from EHRs, the internet, and other public data sources.

While no previous protocol for data collection of this type existed, interview participants mentioned existing processes that could be adapted and applied to the COVID-19 pandemic’s data needs. One interviewee said that the creation of an operational dashboard was facilitated through the preestablished practice of capacity tracking for isolation rooms, negative pressure rooms, and ventilators through their hospital’s EHR system. Other dashboards utilized standardized weekly reporting to keep regional organizations informed on current state resources and utilization.

#### Modeling

Throughout the evolution of COVID-19–related data requests, the need for modeling to project the future number of cases and impact on the state’s health care system remained constant; however, model developers reported that the components and parameters used to model future outcomes evolved substantially, since assumptions were updated as more was learned about COVID-19. Early models were basic and used case counts, though these quickly pivoted to incorporate transmission and disease progression parameters. While NCDHHS primarily uses time-trend modeling for predicting peak surge capacity and informing resource allocation, it has begun partnering with subject-matter experts for predictive modeling [30].

#### Hospital Management

Some interview participants (n=5) described establishing command centers at hospitals to help guide strategic planning. COVID-19 data were used in an operational manner to provide decision support for clinical and administrative executives developing hospital response plans. This included reviewing surveillance reports and inpatient data to monitor positive and negative cases, test volumes, hospitalizations and deaths by age group, and the racial and ethnic breakdown of admissions.

Many hospitals utilized data to predict volumes and develop plans to convert or add hospital space to accommodate COVID-19 patients if needed. Furthermore, interview participants noted how the effective collection and reporting of COVID-19 data meant a hospital would be well-positioned to receive needed allocations of personal protective equipment and treatments.

#### Community Outreach

The importance of transparency and community education was an important theme that arose among interview participants. Webinars and virtual engagements, publications, and televised public service announcements were some of the methods interview participants used to disseminate COVID-19-related information. County school systems, journalists, underserved populations, and local governments and community leaders were among groups targeted by interview participants. One interviewee noted that her group was very cognizant of information overload, contributing to what has been termed *COVID fatigue*, in the general public. In response, they were very intentional when considering what information to release and attempted to tie information to state or local regions to make it more relatable.

### COVID-19 Data Collection Challenges

#### Data Definitions and Consistency

The lack of standardized definitions at the federal level resulted in significant variation in interpreting COVID-19 data within North Carolina. For example, there are several ways organizations can define capacity, and there are different methods for calculating positivity rates. Interview participants made clear their irritation with a lack of clear and consistent definitions across organizations. During interviews, some shared their skepticism surrounding the state’s data quality stemming from the potential for misinterpretation of data or from some groups not being committed to quality control.

#### Collection Process

Participants expressed their frustration with the amount of time needed for COVID-19 data collection. Each new request from the state and federal levels for additional data types required resources to determine what aspects of existing systems needed to be changed or updated. In addition, requests often consisted of continually evolving data requirements and did not take into account the amount of time necessary to adjust established processes to comply with new or modified requests. The ability to meet regulatory requirements was further impacted by a lack of clear authority and defined roles (who to contact for approval of data sharing or to have questions resolved in a timely manner). Many interview participants found themselves unable to access data that they needed and experienced delays caused by waiting for data use agreements. The high number of data requests, changes in data requests, and the urgent nature of these requests led to staff fatigue and burnout. All of these issues proved especially problematic for those working at smaller labs, hospitals, and facilities operating with limited staff and resources.

#### Modeling

Data lags have impacted COVID-19 models, which often require more data to be more accurate. The need for data use agreements has led to frustration among interviewees who were modelers, with one group reporting that if more data had been available to them in the first 90 days or less of building the model, it could have been built faster and more precisely. Others reported now having a better understanding of which information can be requested and shared than they did in March 2020; they therefore request data that does not require a data use agreement. One interview participant remarked that the type of modeling his group has been doing typically takes years and doing so amid a pandemic where information needs are urgent and parameters are constantly changing was a significant added stressor.

#### Contact Tracing

Interview participants cited major obstacles in conducting contact tracing. Since the start of the pandemic, there was an overall increase in the number of cases considered lost to follow up because people were either difficult to reach by phone or unwilling to cooperate with public health officials. For example, interviewees reported that when people were located as part of contact tracing efforts, they seemed reluctant to name who they were in contact with during 2 weeks before symptom onset because those contacts would be required to quarantine. This resulted in a decreasing number of named close contacts among traced individuals. Universities and organizations, mostly health care facilities, were also engaged in contact tracing outside of local health departments. These organizations have trained staff carrying out comprehensive COVID-19 contact tracing plans. Interviewees from some organizations reported carrying out contact tracing for employees only and expressed difficulties in contact tracing outside of their respective institutions.

### Cross-sector Collaboration

A positive byproduct of the COVID-19 pandemic has been the capacity and demand for cross-sector collaboration. Cross-sector collaboration was identified by interviewees as something that North Carolina did very well. Collaborative efforts were mentioned by every interview participant. Some of the groups involved in these collaborations included school systems, government organizations, health systems, pharmaceutical and medical supply companies, think tanks, consulting firms, nonprofit institutions, researchers, educators, health professionals, and foundations. The collaborations were effective in proactively establishing mechanisms to receive state and federal data, facilitating data centralization, and synergizing modeling efforts. On the other hand, the fast-paced and always evolving environment created by COVID-19 was at times difficult to navigate among collaborators. In addition, some interviewees reported there were lost opportunities for collaboration, such as when a lack of awareness of work being done by others resulted in duplicated efforts.

### Technology Integration

Technology plays a critical role in effective data collection and reporting. Several organizations noted success in terms of software or system integrations between the state health department and electronic labs reporting interfaces. Interviewees reported that information technology systems and services were forced to improve or stabilize their products as a byproduct of their data collection and reporting efforts. Furthermore, NCDHHS responded quickly to develop and deploy electronic methods for providers and laboratories to upload data.

## Discussion

### Principal Findings

Through this study, we were able to gather valuable information about COVID-19 data collection and reporting processes from some of the utmost experts and stakeholders in North Carolina. These findings help to inform what happened in North Carolina early in the pandemic, what worked well, and what could be improved.

Interviewees shared a collective goal in serving the people of North Carolina and keeping them informed with up-to-date information that clearly communicated their risk level. The most cited source of COVID-19 data was electronic health records, which was one of several sources utilized to create dashboards. In the United States, all 50 state governments use COVID-19 dashboards that are publicly available. These dashboards contain interactive maps and graphs and report indicators such as deaths, cases, and hospitalizations [31,32]. Widely used during the current pandemic, models have served a number of purposes, including predicting the spread of the virus [33-37] and for evaluating mitigation strategies [38-40]. In North Carolina, COVID-19 data informed the development or adaptation of existing models, which helped forecast the pandemic’s impact on the state’s health care system.

Typically, health care systems and health departments have not used the same software, systems, or data formats, making it difficult to identify trends during outbreaks and develop mitigation strategies [41]. Key informants reported success in integrating and revising multiple data collection systems, and NCDHHS provided timely guidance to stakeholders who upload COVID-19 data. System integration can play a pivotal role in the success of reporting data during future pandemics, and public health infrastructure would benefit from additional funding for data-related health information technology projects at state and federal levels. Innovative integrated technologies would help public health researchers, health care workers, and government officials remain connected, by providing data that is needed to understand outbreaks and coordinate responses.

Interviewees faced a number of challenges when collecting and using COVID-19 data. At the root of these issues was the fast pace at which knowledge about the virus evolved. This directly affected the type of data requested from state and federal governments and turnaround time for submission. Further exacerbating these issues was a lack of standardized data definitions and defined roles (who to contact when clarification was needed). This experience was not unique to North Carolina, but rather common among research institutes where a lack of time led to an inability to coordinate data standardization and define and share vocabularies, which slowed or prevented the ability to collaborate and share data [42].

Interviewees reported that the pervasive sense of urgency and need to collect and report the most accurate data possible led to significant stress and burnout among staff participating in these efforts. This finding is in alignment with those from a study [43] of public health workers who worked in state, local, tribal, or territorial health departments during 2020. When asked about the preceding 2 weeks, 53% reported experiencing symptoms of at least 1 mental health condition (depression, anxiety, posttraumatic stress disorder, or suicidal ideation) and 72% had felt overwhelmed by workload or family–work balance. Fortunately, interviewees in our study described a strong support system that emerged in North Carolina from the cross-sector collaboration of those involved in data collection. These partnerships allowed them to synergize efforts to identify issues and work together to proffer solutions. Guiding these efforts was the strong leadership from NCDHHS which provided much needed support throughout the entire process.

Our findings provide insight that can be used to inform the state responses to future public health emergencies. Based on the findings of this study, we compiled the following lessons learned for North Carolina to improve pandemic response and better prepare for future public health crises.

Future pandemic response requires centralization through the North Carolina Department of Health and Human Services. Standardized and coordinated information sharing is the foundation of effective pandemic response. Interview participants voiced their appreciation for the leadership exemplified by NCDHHS following the COVID-19 outbreak and a desire for streamlined processes when preparing for and responding to future pandemics. They expressed frustration over requirements imposed by the federal government that were made without appropriate guidance and with very short timelines for compliance. Interview participants emphatically asserted that, even in such cases, the leadership and coordination provided by NCDHHS helped alleviate the difficult circumstances.

Cross-sector collaborative networks established during the COVID-19 outbreak should be supported and sustained. Cross-sector collaboration was a consistent theme mentioned by key informants, who considered it a major facilitator in the collection and use of COVID-19–related data. Many of these collaborations developed from existing relationships and a desire to maximize the combined impact of the work being performed by colleagues at different institutions. North Carolina is fortunate to have a number of strong research institutes and would benefit from formalizing many of the collaborative networks that have organically developed since March 2020. In supporting these partnerships, and defining the roles of each team member, the state could encourage even more data synergy and consistency in data collection processes moving forward.

Pandemic-related data should be publicly accessible and available in a format that is easy to use and understand, such as real-time dashboards. As was the case with COVID-19, pandemic response can result in frequent changes to data and surveillance systems, which may not always be well explained, leading to public and provider mistrust. Data transparency via open access can build trust during outbreaks and encourage public adherence to disease prevention and control mandates [44]. Proactive data collection and analysis facilitate identification of patterns and timely dissemination of information. To increase access, North Carolina should release data in an easy-to-download format to not only inform the public but also to facilitate analysis by data scientists. Open and accessible data sharing can promote collaboration among scientists, public health professionals, and lawmakers and inform policies and interventions to mitigate future outbreaks. Furthermore, data should be translated in a manner useful to the greater public, by using summaries and highlighting key messages [45]. Alternatively, health departments could create a public version of future dashboards that contain information and metrics specifically considered to be of value to the public [46].

### Limitations

We note several limitations in this study. The main limitation is that qualitative research does not provide generalizability. Nor does it provide statistical representation of larger populations. While we have obtained and summarized common themes expressed among interview participants, these themes cannot be generalized to the larger population of North Carolina. The information presented here is descriptive and meant to provide insight into the experiences and opinions of stakeholders represented by the sample population. Additionally, in recruiting interviewees, we were unable to obtain participation from city or county public health workers. At the time of recruitment, the state health department reported that not all counties had the capacity to collect data, and there was no comprehensive list of county-level data collection. Because surveillance data were being aggregated at the state level, we decided to collect data from state health department workers. Furthermore, due to the rapid evolution of the pandemic, there was an urgency to disseminate the results of this study as quickly as possible to inform data collection efforts in North Carolina. We, therefore, were unable to address some of these limitations. Future research may be helpful to understand the successes or challenges experienced by city and county health department workers in North Carolina during the early phases of the COVID-19 pandemic.

### Conclusion

The fast-paced nature of the COVID-19 pandemic has required an agile response from those collecting and using COVID-19 data to inform preparation and response at national, state, and local levels. Study results show the importance of data flow in a pandemic, the value of dashboards and modeling in decision-making, and the vital role of cross-sector collaboration. It is important to note that the experiences and challenges of key informants were likely not exclusive to North Carolina; however, stakeholders benefited from the strong leadership of the state health department in coordinating data collection and reporting. As the state moves closer to having the majority of the population vaccinated, and ideally, herd immunity, we look optimistically toward a new normal in a post–COVID-19 era. Nonetheless, more pandemics are inevitable, and successful preparedness can increase readiness and the ability to react swiftly. This study’s results can be used to build on ongoing pandemic-related work and help develop a strong nationally coordinated approach to data collection, reporting, dissemination, and intercommunication among stakeholders.

## Abbreviations

EHR: electronic health record
NCDHHS: North Carolina Department of Health and Human Services

## References

[1] (2020). COVID-19 public health emergency of international concern (PHEIC). World Health Organization.

[2] Dong E, Du H, Gardner L (2020). An interactive web-based dashboard to track COVID-19 in real time. Lancet Infect Dis.

[3] Unwin HJT, Mishra S, Bradley VC, Gandy A, Mellan TA, Coupland H, Ish-Horowicz J, Vollmer MAC, Whittaker C, Filippi SL, Xi X, Monod M, Ratmann O, Hutchinson M, Valka F, Zhu H, Hawryluk I, Milton P, Ainslie KEC, Baguelin M, Boonyasiri A, Brazeau NF, Cattarino L, Cucunuba Z, Cuomo-Dannenburg G, Dorigatti I, Eales OD, Eaton JW, van Elsland SL, FitzJohn RG, Gaythorpe KAM, Green W, Hinsley W, Jeffrey B, Knock E, Laydon DJ, Lees J, Nedjati-Gilani G, Nouvellet P, Okell L, Parag KV, Siveroni I, Thompson HA, Walker P, Walters CE, Watson OJ, Whittles LK, Ghani AC, Ferguson NM, Riley S, Donnelly CA, Bhatt S, Flaxman S (2020). State-level tracking of COVID-19 in the United States. Nat Commun.

[4] Srinivasan A, McDonald LC, Jernigan D, Helfand R, Ginsheimer K, Jernigan J, Chiarello L, Chinn R, Parashar U, Anderson L, Cardo D, SARS Healthcare Preparedness Response Plan Team (2004). Foundations of the severe acute respiratory syndrome preparedness and response plan for healthcare facilities. Infect Control Hosp Epidemiol.

[5] (2004). Public health guidance for community-level preparedness and response to severe acute respiratory syndrome (SARS). Centers for Disease Control and Prevention.

[6] Savin-Baden M, Howell-Major C (2013). Qualitative Research: The Essential Guide to Theory and Practice.

[7] Matua GA, Van Der Wal DM (2015). Differentiating between descriptive and interpretive phenomenological research approaches. Nurse Res.

[8] Hunter DJ, McCallum J, Howes D (2019). Defining Exploratory-Descriptive Qualitative (EDQ) research and considering its application to healthcare. J Nurs Healthc.

[9] Tyrer S, Heyman B (2016). Sampling in epidemiological research: issues, hazards and pitfalls. BJPsych Bull.

[10] Etikan I, Alkassim R, Abubakar S (2016). Comparision of snowball sampling and sequential sampling sechnique. Biom Biostat Int J.

[11] Moser A, Korstjens I (2018). Series: Practical guidance to qualitative research. part 3: Sampling, data collection and analysis. Eur J Gen Pract.

[12] Isaacs A (2014). An overview of qualitative research methodology for public health researchers. Int J Med Public Health.

[13] Karnieli-Miller O, Strier R, Pessach L (2009). Power relations in qualitative research. Qual Health Res.

[14] Newcomer K, Hatry H, Wholey J (2015). Handbook of Practical Program Evaluation, 4th ed.

[15] Bengtsson M (2016). How to plan and perform a qualitative study using content analysis. NursingPlus Open.

[16] Cho J, Lee E (2014). Reducing confusion about grounded theory and qualitative content analysis: similarities and differences. Qual Rep.

[17] Lindgren B, Lundman B, Graneheim UH (2020). Abstraction and interpretation during the qualitative content analysis process. Int J Nurs Stud.

[18] Elliott V (2018). Thinking about the coding process in qualitative data analysis. Qual Rep.

[19] Ryan GW, Bernard HR (2003). Techniques to identify themes. Field Methods.

[20] Korstjens I, Moser A (2018). Series: Practical guidance to qualitative research. part 4: trustworthiness and publishing. Eur J Gen Pract.

[21] Fusch P, Fusch GE, Ness LR (2018). Denzin’s paradigm shift: revisiting triangulation in qualitative research. J Soc Change.

[22] (2020). NCDHHS guidance for reporting of COVID-19 diagnostic test results. North Carolina Department of Health and Human Services.

[23] North Carolina Department of Health and Human Services, University of North Carolina School of Medicine NC DETECT. North Carolina Department of Health and Human Services.

[24] (2021). The New York Times COVID-19-data. GitHub.

[25] (2021). Coronavirus. The News & Observer.

[26] (2021). NC coronavirus updates and latest news. WRAL.

[27] Majumder M, Rose S (2020). Health Affairs. Health care claims data may be useful for COVID-19 research despite significant limitations.

[28] (2021). Safegraph. Safegraph.

[29] (2021). PolicyMap. PolicyMap.

[30] North Carolina COVID-19 modeling shows social distancing necessary to slow the spread and preserve hospital capacity to save lives. North Carolina Department of Health and Human Services.

[31] Fareed N, Swoboda CM, Chen S, Potter E, Wu DTY, Sieck CJ (2021). U.S. COVID-19 state government public dashboards: an expert review. Appl Clin Inform.

[32] Zylla E, Hartman L (2020). State COVID-19 data dashboards. State Health and Value Strategies.

[33] Wu JT, Leung K, Leung GM (2020). Nowcasting and forecasting the potential domestic and international spread of the 2019-nCoV outbreak originating in Wuhan, China: a modelling study. Lancet.

[34] Park SW, Cornforth DM, Dushoff J, Weitz JS (2020). The time scale of asymptomatic transmission affects estimates of epidemic potential in the COVID-19 outbreak. Epidemics.

[35] Li R, Pei S, Chen B, Song Y, Zhang T, Yang W, Shaman J (2020). Substantial undocumented infection facilitates the rapid dissemination of novel coronavirus (SARS-CoV2). Science.

[36] Kucharski AJ, Russell TW, Diamond C, Liu Y, Edmunds J, Funk S, Eggo RM, Centre for Mathematical Modelling of Infectious Diseases COVID-19 working group (2020). Early dynamics of transmission and control of COVID-19: a mathematical modelling study. Lancet Infect Dis.

[37] Li L, Yang Z, Dang Z, Meng C, Huang J, Meng H, Wang D, Chen G, Zhang J, Peng H, Shao Y (2020). Propagation analysis and prediction of the COVID-19. Infect Dis Model.

[38] Koo JR, Cook AR, Park M, Sun Y, Sun H, Lim JT, Tam C, Dickens BL (2020). Interventions to mitigate early spread of SARS-CoV-2 in Singapore: a modelling study. Lancet Infect Dis.

[39] Pan A, Liu L, Wang C, Guo H, Hao X, Wang Q, Huang J, He N, Yu H, Lin X, Wei S, Wu T (2020). Association of public health interventions with the epidemiology of the COVID-19 outbreak in Wuhan, China. JAMA.

[40] Prem K, Liu Y, Russell TW, Kucharski AJ, Eggo RM, Davies N, Jit M, Klepac P, Centre for the Mathematical Modelling of Infectious Diseases COVID-19 Working Group (2020). The effect of control strategies to reduce social mixing on outcomes of the COVID-19 epidemic in Wuhan, China: a modelling study. Lancet Public Health.

[41] He W, Zhang ZJ, Li W (2021). Information technology solutions, challenges, and suggestions for tackling the COVID-19 pandemic. Int J Inf Manage.

[42] Dagliati A, Malovini A, Tibollo V, Bellazzi R (2021). Health informatics and EHR to support clinical research in the COVID-19 pandemic: an overview. Brief Bioinform.

[43] Bryant-Genevier J, Rao CY, Lopes-Cardozo B, Kone A, Rose C, Thomas I, Orquiola D, Lynfield R, Shah D, Freeman L, Becker S, Williams A, Gould DW, Tiesman H, Lloyd G, Hill L, Byrkit R (2021). Symptoms of Depression, Anxiety, Post-Traumatic Stress Disorder, and Suicidal Ideation Among State, Tribal, Local, and Territorial Public Health Workers During the COVID-19 Pandemic - United States, March-April 2021. MMWR Morb Mortal Wkly Rep.

[44] Finset A, Bosworth H, Butow P, Gulbrandsen P, Hulsman RL, Pieterse AH, Street R, Tschoetschel R, van Weert J (2020). Effective health communication - a key factor in fighting the COVID-19 pandemic. Patient Educ Couns.

[45] Huston P, Edge VL, Bernier E (2019). Reaping the benefits of open data in public health. Can Commun Dis Rep.

[46] Dixon BE, Grannis SJ, McAndrews C, Broyles AA, Mikels-Carrasco W, Wiensch A, Williams JL, Tachinardi U, Embi PJ (2021). Leveraging data visualization and a statewide health information exchange to support COVID-19 surveillance and response: application of public health informatics. J Am Med Inform Assoc.

